# Fire Ant Decapitating Fly Cooperative Release Programs (1994–2008): Two *Pseudacteon* Species, *P. tricuspis* and *P. curvatus*, Rapidly Expand Across Imported Fire Ant Populations in the Southeastern United States

**DOI:** 10.1673/031.011.0119

**Published:** 2011-02-18

**Authors:** Anne-Marie A. Callcott, Sanford D. Porter, Ronald D. Weeks, L. C. “Fudd” Graham, Seth J. Johnson, Lawrence E. Gilbert

**Affiliations:** ^1^ USDA, APHIS, PPQ, Center for Plant Health Science and Technology, Gulfport Laboratory, 3505 25th Avenue, Gulfport, MS 39501; ^2^ Center for Medical, Agricultural and Veterinary Entomology, USDA-ARS, 1600 SW 23rd Drive, Gainesville, FL 32608; ^3^ USDA, APHIS, PPQ, Eastern Region Office, 920 Main Campus Drive, Raleigh, NC 27606-5213; ^4^ Department of Entomology and Plant Pathology, Auburn University, 301 Funchess Hall, Auburn, AL 36849-5413; ^5^ Department of Entomology, 400 Life Sciences Building, Louisiana State University Agricultural Center, Baton Rouge, LA 70803; ^6^ Brackenridge Field Laboratory and Section of Integrative Biology, The University of Texas, Austin, TX 78712

**Keywords:** biocontrol, biological control, distribution, expansion, Formicidae, Phoridae, *Solenopsis invicta*, *Solenopsis richteri*

## Abstract

Natural enemies of the imported fire ants, *Solenopsis invicta* Buren *S. richteri* Forel (Hymenoptera: Formicidae), and their hybrid, include a suite of more than 20 fire ant decapitating phorid flies from South America in the genus *Pseudacteon*. Over the past 12 years, many researchers and associates have cooperated in introducing several species as classical or self-sustaining biological control agents in the United States. As a result, two species of flies, *Pseudacteon tricuspis* Borgmeier and *P. curvatus* Borgmeier (Diptera: Phoridae), are well established across large areas of the southeastern United States. Whereas many researchers have published local and state information about the establishment and spread of these flies, here distribution data from both published and unpublished sources has been compiled for the entire United States with the goal of presenting confirmed and probable distributions as of the fall of 2008. Documented rates of expansion were also used to predict the distribution of these flies three years later in the fall of 2011. In the fall of 2008, eleven years after the first successful release, we estimate that *P. tricuspis* covered about 50% of the fire ant quarantined area and that it will occur in almost 65% of the quarantine area by 2011. Complete coverage of the fire ant quarantined area will be delayed or limited by this species' slow rate of spread and frequent failure to establish in more northerly portions of the fire ant range and also, perhaps, by its preference for red imported fire ants (*S. invicta*). Eight years after the first successful release of *P. curvatus*, two biotypes of this species (one biotype occurring predominantly in the black and hybrid imported fire ants and the other occurring in red imported fire ants) covered almost 60% of the fire ant quarantined area. We estimate these two biotypes will cover almost 90% of the quarantine area by 2011 and 100% by 2012 or 2013. Strategic selection of several distributional gaps for future releases will accelerate complete coverage of quarantine areas. However, some gaps may be best used for the release of additional species of decapitating flies because establishment rates may be higher in areas without competing species.

## Introduction

Imported fire ants, *Solenopsis invicta* Buren and *Solenopsis richteri* Forel (Hymenoptera: Formicidae), invaded the United States more than 75 years ago. Today they have expanded their range into at least 13 states and Puerto Rico and over 1,385,000 km^2^ (Callcott and Collins 1996; updated by A-M.A.C.). While *S. invicta* is present throughout most of the southeastern United States, *S. richteri* and the *S. invicta x S. richteri* hybrid are confined to northern areas of Mississippi (Streett et al. 2006), Alabama (Bertagnolli et al. 2007), Georgia (Gardner et al. 2008), and the southern two-thirds of Tennessee (Oliver et al. 2009).

Imported fire ants are 5–10 times more abundant in the United States than they are in their South American homeland (Porter et al. 1992; Porter et al. 1997). Escape from natural biological control agents left behind in South America is the most likely explanation for these intercontinental differences in fire ant populations. Consequently, it is possible that the introduction of biocontrol agents from South America may help tip the ecological balance in favor of native ants (Porter 1998) and reduce North American fire ant populations to levels more like those found in South America (Porter et al. 1997).

Natural enemies of imported fire ants include several microsporidian pathogens (Jouvenaz 1983; Oi and Williams 2002; Briano 2005; Oi and Valles 2008), at least three newly discovered viruses (Valles et al. 2007a; Valles et al. 2007b; Valles and Hashimoto 2009), several kinds of nematodes (Jouvenaz et al. 1988; Poinar et al. 2007), and a variety of arthropod parasites and parasitoids (Wojcik 1989; Wojcik et al. 1991; Calcaterra et al. 2001; Varone and Briano 2009) including at least 24 species of very small phorid flies in the genus *Pseudacteon* (Porter and Pesquero 2001; Brown et al. 2003; Calcaterra 2007; Kronforst et al. 2007; Patrock et al. 2009). Flies in the genus *Pseudacteon* (Diptera: Phoridae) are known as decapitating flies because their larvae have the unusual habit of decapitating their host and then using the empty ant head capsule as a pupal case (Porter et al. 1995a; Consoli et al. 2001). Different species partition niche space by host size, season, time of day, and mode of attack (Campiolo et al. 1994; Fowler et al. 1995; Pesquero et al. 1996; Morrison et al. 1997; Orr et al. 1997; Folgarait et al. 2003). Certain fly species or biotypes prefer specific fire ant hosts (i.e. black imported fire ants, red imported fire ants, or their hybrid; Porter and Briano 2000; Vazquez et al. 2006). Consequently, the introduction of a selection of several fly species is expected to be necessary to have maximum impact on fire ant populations.

The decapitating flies *Pseudacteon tricuspis* Borgmeier and *P. curvatus* Borgmeier (Diptera: Phoridae) were first successfully released in the United States beginning in 1997 (Porter et al. 2004) and 2000 (Graham et al. 2003b) as self-sustaining, classical biological control agents of the imported fire ants: *S. invicta, S. richteri*, and their hybrid. A third phorid species, *Pseudacteon litoralis* Borgmeier, was established at a single site in Alabama in 2005 (Porter et al. 2011) and a fourth species, *Pseudacteon obtusus* Borgmeier, has been established recently at sites in Texas (Gilbert et al. 2008) and Florida (S.D.P.). *Pseudacteon cultellatus* Borgmeier has recently been approved for field release (April 2010) and *Pseudacteon nocens* Borgmeier and several additional fly species are being reared and/or test-released in Austin, TX (L.E.G.).

In the past 10+ years, a great deal of research has been conducted with fire ant decapitating flies and a large effort has been expended to establish them in the United States. Research has addressed aspects of basic biology (Porter 1998; Morrison 2000), ecology (Feener 2000; Morrison et al. 2000; Folgarait et al. 2005), taxonomy (Porter and Pesquero 2001), behavior (Orr et al. 1995; Orr et al. 1996; Wuellner et al. 2002), host specificity (Porter and Gilbert 2004), and automated rearing techniques (Vogt et al. 2003). Numerous studies on the establishment and spread of the phorid flies have been published (Table 1). However, all of these studies have focused on local or state levels. Since the overall distribution of *P. tricuspis* and *P. curvatus* in the United States has not yet been compiled, an attempt was made to gather distribution data available from various sources into one publication. The primary objective of this study is to show the confirmed and probable distributions of *P. tricuspis* and *P. curvatus* in the southeastern United States as of the fall of 2008. Data on where and when these flies were released are also provided. Rates of expansion were observed to predict where these flies will be by the fall of 2011 and when they are likely to achieve complete coverage of fire ant populations in the United States.

### History of rearing and release programs

In 1994, two independent projects were initiated in Brazil with the goal of studying *Pseudacteon* phorid flies for biological control of invasive *Solenopsis* fire ants. The USDA-ARS fire ant research project (Gainesville, FL) focused on phorid fly life history studies and rearing methods. These studies were conducted in collaboration with Harold Fowler at Sao Paulo State University in Rio Claro and Luiz Alexandre Nogueira de Sa at the Embrapa research center near Jaguariuna, SP, Brazil. The University of Texas project (Austin, TX) focused on ecological studies of fire ants and phorid flies. Their efforts in Brazil were conducted in cooperation with Woodruff Benson at the University of Campinas, Brazil.

The University of Texas project obtained permits to release four species of South American *Pseudacteon* decapitating flies in the United States in May 1995 (Gilbert and Patrock 2002); however, early releases associated with this permit were not successful probably because of problems related to rearing, droughts, and the small numbers of flies available for release (Gilbert et al. 2008). The University of Texas group then began cooperating with Patricia Folgarait (Universidad Nacional de Quilmes, Buenos Aires, Argentina) and focused on rearing and releasing *P. tricuspis* and *P. curvatus* in the hot and drier eco-regions of central and southern Texas (Appendices 1, 2; Gilbert and Patrock 2002; Gilbert et al. 2008).

The USDA-ARS project obtained authorization to release *P. tricuspis* in 1997. Favorable climatic conditions in Florida and the ability to rear and release several thousand flies from laboratory colonies resulted in at least five successful releases around Gainesville, FL between 1997 and 1999 (Porter et al. 2004). The USDA-ARS obtained permission to field release *P. curvatus* in 2000. After releases in Florida, Alabama, and Tennessee (2000–2001), this colony was transferred to the USDA-ARS BCPRU laboratory in Starkville, MS that used the flies for releases in Clay County, MS (Vogt et al. 2003) and additional releases in the three states just mentioned. In 2001, a second biotype of *P. curvatus* was collected in Argentina and successfully released against red imported fire ants at three sites near Gainesville in 2003 (Vazquez et al. 2006). All together, USDA-ARS and their state cooperators released *P. tricuspis* in 11 states and *P. curvatus* flies in six states between 1997 and 2004 (Appendices 1 and 2).

In 2001, the USDA-APHIS initiated a cooperative program to rear and release decapitating flies as fire ant biocontrol agents in all infested states (Callcott et al. 2007). The Florida Department of Agriculture, Division of Plant Industry (FL-DPI), in Gainesville, Florida was contracted to mass-rear the phorid flies and distribute them for release. USDA-ARS (Gainesville, FL) assisted by collecting and evaluating new fly species for potential field release, establishing the initial laboratory colonies of flies. USDA-APHIS coordinated releases with state cooperators in each of the fire ant-infested states including Puerto Rico (Table 1; Appendices 1, 2), who in turn released the flies and monitored establishment and spread. The USDA-APHIS program began in 2002 with *P. tricuspis* and in 2004 with *P. curvatus*. Louisiana State University also successfully reared *P. tricuspis* flies obtained from the USDA-APHIS program and used these flies for two releases in Louisiana (Table 1; Appendix 1).

The USDA-ARS laboratories in Florida and Mississippi and their cooperators released *P. tricuspis* at one or more sites in 27 counties and *P. curvatus* at one or more sites in 13 counties across the southern United States from 1997–2004 (Appendices 1, 2). Between 1995 and 2008, the University of Texas laboratory and their cooperators released *P. tricuspis* at one or more sites in 21 counties and *P. curvatus* at one or more sites in 14 counties, with both species released in some counties (Appendices 1, 2). Between 2001 and 2008, the USDA-APHIS cooperative program provided *P. tricuspis* and *P. curvatus* flies for one or more releases in 45 and 31 counties, respectively, across the southern U.S. (including Puerto Rico) and California (Appendices 1, 2). Overall, from 1995 to 2008, *P. tricuspis* and *P. curvatus* were released in 114 counties in the United States. One or more releases have occurred in 32 Texas counties, 14 Alabama counties, and 10 or fewer counties in each of the remaining southern states, Puerto Rico, and California.

### Sources of flies

All of the *P. tricuspis* flies released in the United States outside of Texas were collected by S.D.P. from the Jaguariuna Embrapa research center just north of Campinas, SP, Brazil (Appendix 1). Most of the flies released in east Texas and north Texas were also Jaguariuna flies. A biotype of *P. tricuspis* collected by S.D.P. and Juan Briano from a site 35 km NW of Formosa, Argentina in October 2001 (see Vazquez et al. 2006) was released at the USDA-ARS Areawide research site north of Caldwell, Texas (Burleson Co.) in the spring of 2003 (Barr and Calixto 2004). Flies released by the University of Texas in central and south Texas were from a mixture of locations including flies they had collected in Campinas, Brazil supplemented by the two biotypes mentioned above (Gilbert et al. 2008).

Two biotypes of *P. curvatus* have been released in the United States. The first was collected from S. richteri (black fire ants) near Las Flores, Buenos Aires, Argentina by S.D.P. with the help of Juan Briano (Graham et al. 2003b). When the Las Flores biotype was released on red imported fire ants in the United States around Gainesville, Florida (2000–2001), it failed to establish seven times (Graham et al. 2003b; not shown in Appendix 2). A small release of the Las Flores biotype in Oklahoma on red fire ants in 2002 probably also failed. However, almost every release on black and hybrid fire ants in Tennessee, Mississippi, and Alabama succeeded (Graham et al. 2003b; Vogt and Streett 2003; Parkman et al. 2005; Appendix 2).

A second biotype of *P. curvatus* was collected from red fire ants at a site 35 km NW of Formosa, Argentina by S.D.P. and Juan Briano (Vazquez et al. 2006). The first releases of the Formosa biotype were made in 2003 around Gainesville, FL (Vazquez et al. 2006). All of the *P. curvatus* releases after 2003 in Arkansas, Florida, Georgia, Louisiana, North Carolina, Oklahoma, Puerto Rico, South Carolina, and Texas were Formosa biotype flies (Appendix 2). A population of the Formosa biotype initiated at University of Texas' Brackenridge Field Laboratory in 2004, with less than 200 female flies from the Gainesville colony, was the foundation for the release and spread of *P. curvatus* across much of central and southern Texas.

### Assessment of impacts

Fire ants have evolved a suite of defensive behaviors to protect them against *Pseudacteon* decapitating flies (Porter 1998). Worker ants are keenly aware of phorid flies and the presence of even a single fly can stop or greatly inhibit the foraging efforts of hundreds of workers (Feener and Brown 1992; Orr et al. 1995; Porter et al. 1995b). Reduced foraging appears to facilitate competition from ants that might otherwise be excluded from food sources in fire ant territories (Feener and Brown 1992; Orr et al. 1995; Porter et al. 1995b; Morrison 1999; Mehdiabadi and Gilbert 2002). Workers may hide, remain motionless, or curl into an upside down “C” posture that appears to protect them from parasitism.

Morrison and Porter (2005a) reported that the impacts of a single species of fly, *P. tricuspis*, on fire ant populations in north central Florida did not rise above background variability (10–30%). Several other studies have indicated negative impacts, but sample sizes have been small (Graham et al. 2003a; Vander Meer et al. 2007; Oi et al. 2008). Fire ant populations in the United States are unusually high compared with populations in their native range (Porter et al. 1997). Are phorid decapitating flies the reason? Probably not, in and of themselves, but they almost certainly contribute to this intercontinental difference by 1) killing a small percentage of workers directly (Morrison and Porter 2005b), 2) limiting foraging during daylight hours (Porter 1998), and perhaps 3) vectoring pathogens among colonies (Oi et al. 2009). It is important to note that decapitating flies and other natural enemies do not need to kill fire ant colonies directly to provide benefits. If they stress colonies enough that native ants can compete more effectively, they may tilt the ecological balance in favor of native species.

A number of studies are either underway or planned to assess impacts of flies on fire ants in the U.S. Additional studies are planned to determine field parasitism rates of *Pseudacteon* flies seasonally at several locations near Gainesville, FL using methods similar to those Morrison and Porter (2005b) used for *P. tricuspis*. The potential of *Pseudacteon* flies to vector known fire ant pathogens is also being investigated. Recent studies indicate that *Pseudacteon* flies probably do not vector the virus, SINV-1, or the microsporidian pathogen, *Vairimorpha invictae* (Valles and Porter 2007; Oi et al. 2009). However, *Pseudacteon* flies are carriers of the fire ant microsporidian pathogen *Kneallhazia solenopsae* (Oi et al. 2009), and tests are in progress to determine if the flies can vector this disease among colonies. Studies underway in Texas will compare fire ant populations before and after the releases of phorid flies. To date, the results have been ambiguous, at least partly because extreme drought conditions have disrupted fire ant populations at many of the sites. Additional studies in Louisiana will seek to determine the impact of phorids on the number and size of mounds.

### Field releases and monitoring

Release techniques varied according to fly species and the organization conducting the releases. They also evolved through time, but generally involved either releasing adult flies over active fire ant mounds (*P. tricuspis*, Porter et al. 2004), or shipping worker ants to the lab for parasitism and returning them to their original colony (*P. curvatus*, Graham et al. 2003b; Vogt and Streett 2003; Vazquez et al. 2006; Gilbert et al. 2008). Sometimes, both techniques were used.

The USDA-APHIS program shipped pupae for *P. tricuspis* releases and worker ants for *P. curvatus* releases by commercial carrier to state cooperators who then hand carried adult flies or worker ants to the field. The University of Texas project primarily hand delivered pupae to cooperators or hand-carried adult flies or worker ants from the lab to release sites. The USDA-ARS both hand delivered and commercially shipped pupae or worker ants to cooperators. Thus data from different sources varied according on the numbers of pupae shipped, adult flies released, grams of worker ants introduced into lab colonies for parasitization, and number of mounds used to obtain worker ants. Based on available emergence data, pupae shipped by commercial carrier had significantly lower emergence rates than pupae hand carried to cooperators (ca. 50% vs. 75%, respectively). The USDA-APHIS program used a standard calculation to determine the approximate number of potential flies shipped to the field when worker ants were shipped to the lab for parasitization with *P. curvatus* regardless of the number of mounds where ants were collected: weight of ants (g) × 800 ants/g × 0.30 (ca. 30% parasitization rate). To standardize University of Texas data, especially with *P. curvatus* releases that cite number of mounds, a similar calculation as above was used, estimating that 2 g of workers would be collected from each mound (Gilbert et al. 2008), but with 575 ants/g and a 16% average parasitism rate (R. Plowes, personal communication).

Generally, a release event in the USDA-APHIS program consisted of multiple releases over 2–4 weeks at one site and involved 2–20 thousand flies, or potential flies, with smaller numbers for *P. tricuspis* (3,400/release) and larger numbers for *P. curvatus* (15,300/release). USDA-ARS also conducted multiple releases over a 2–4 week period at one site using ca. 2500/release for *P. tricuspis* and 14,500/release for *P. curvatus*. University of Texas conducted multiple releases over longer periods of several months to several years at one site, resulting in 2–30 thousand flies or potential flies being released at one site with 8,850/release for *P. tricuspis* and 6,800/release for *P. curvatus*.

Flies were considered to be established at a site after they were observed to overwinter and expand their distribution locally. Monitoring of establishment and spread was originally done by disturbing fire ant mounds and visually looking for the flies (Porter et al. 2004). This method is labor intensive and tedious. Puckett et al. (2007) developed a phorid fly trap that successfully monitored establishment and spread of the flies and allowed states with limited staff to survey more sites in a shorter time. The University of Texas monitored phorid flies with a trap using sticky fly paper that allowed rapid scoring and long term storage of specimens (LeBrun et al. 2008). Data presented here were collected either by these three methods, or by methods noted in references cited.

**Figure 1.**
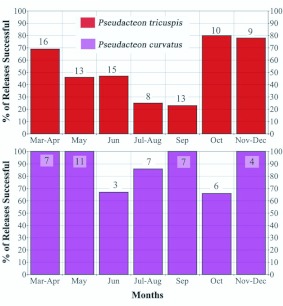
Percent of *Pseudacteon tricuspis* (top, N = 84) and *Pseudacteon curvatus* (bottom, N = 45) releases successful as a function of month for all releases occurring over a 1–4 week period. Numbers on or over bars indicate the number of release sites for that period. Months with 2 or fewer releases were combined with adjacent months. High quality figures are available online.

Establishment of *P. tricuspis* was 51% successful at sites where releases were conducted over 2–3 weeks, and 59% successful where releases occurred over months or years at one site (Appendix 1). For *P. curvatus*, shorter term release events resulted in an 89% success rate and longer term release events resulted in a 100% success rate (Appendix 2). The success of *P. tricuspis* releases in the months of May through September was significantly less than that of releases made earlier in spring and later in fall (χ^2^ = 11.5, 1 df, P < 0.005; Figure 1). The success rate of *P. curvatus* releases was not significantly related to season because only two of the four failed releases occurred in the summer. No relationship was found between the number of flies released and success rate for either *P. tricuspis* or *P. curvatus* (two-tailed t-Tests; P ≥ 0.2).

### Estimating fly distributions

**Figure 2.**
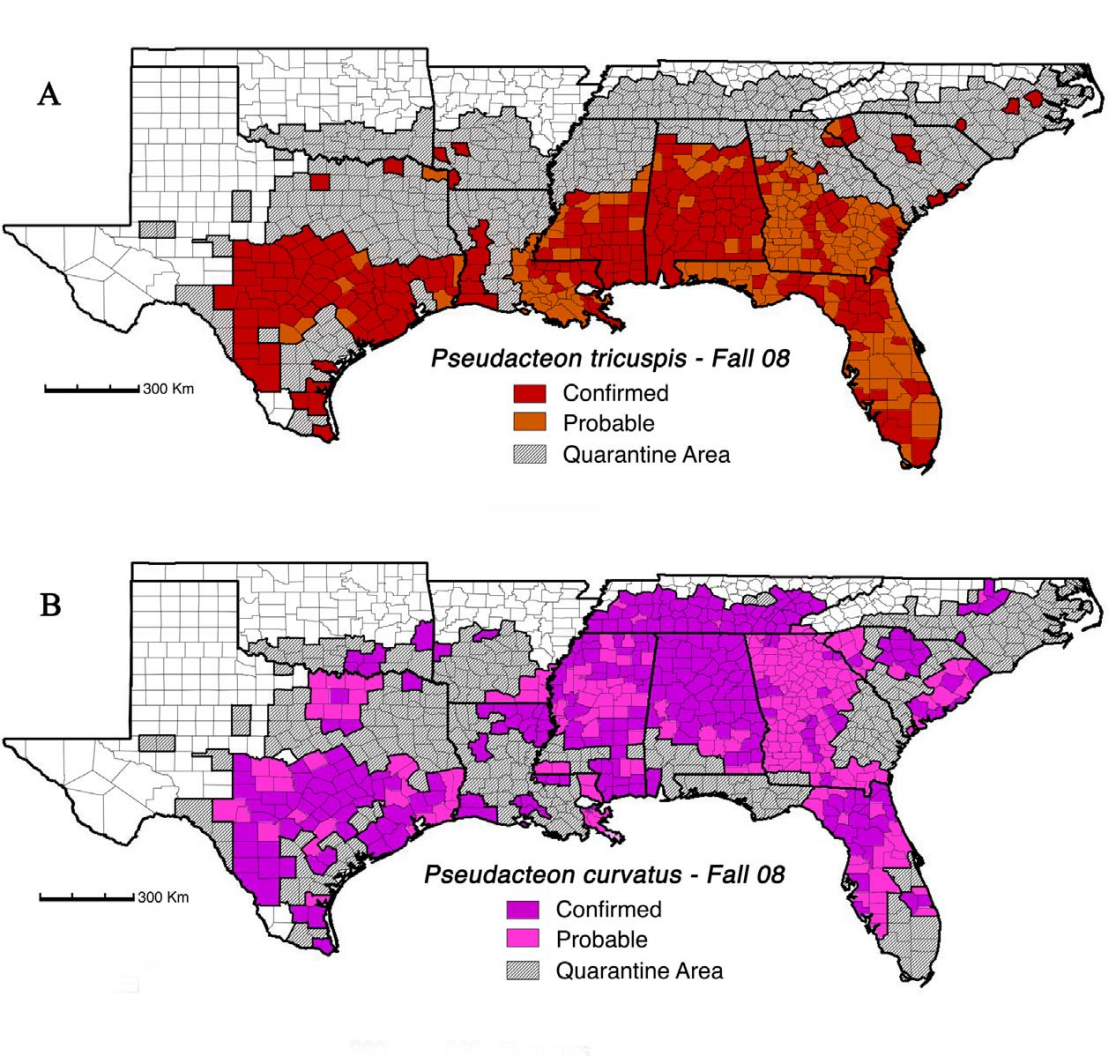
Confirmed and probable distributions of the fire ant decapitating flies, *Pseudacteon tricuspis* (A) and *Pseudacteon curvatus* (B) in counties of 11 southeastern states in the fall of 2008. Counties in the federal fire ant quarantine area (spring 2009) are indicated by grey diagonal lines. High quality figures are available online.

**Figure 3.**
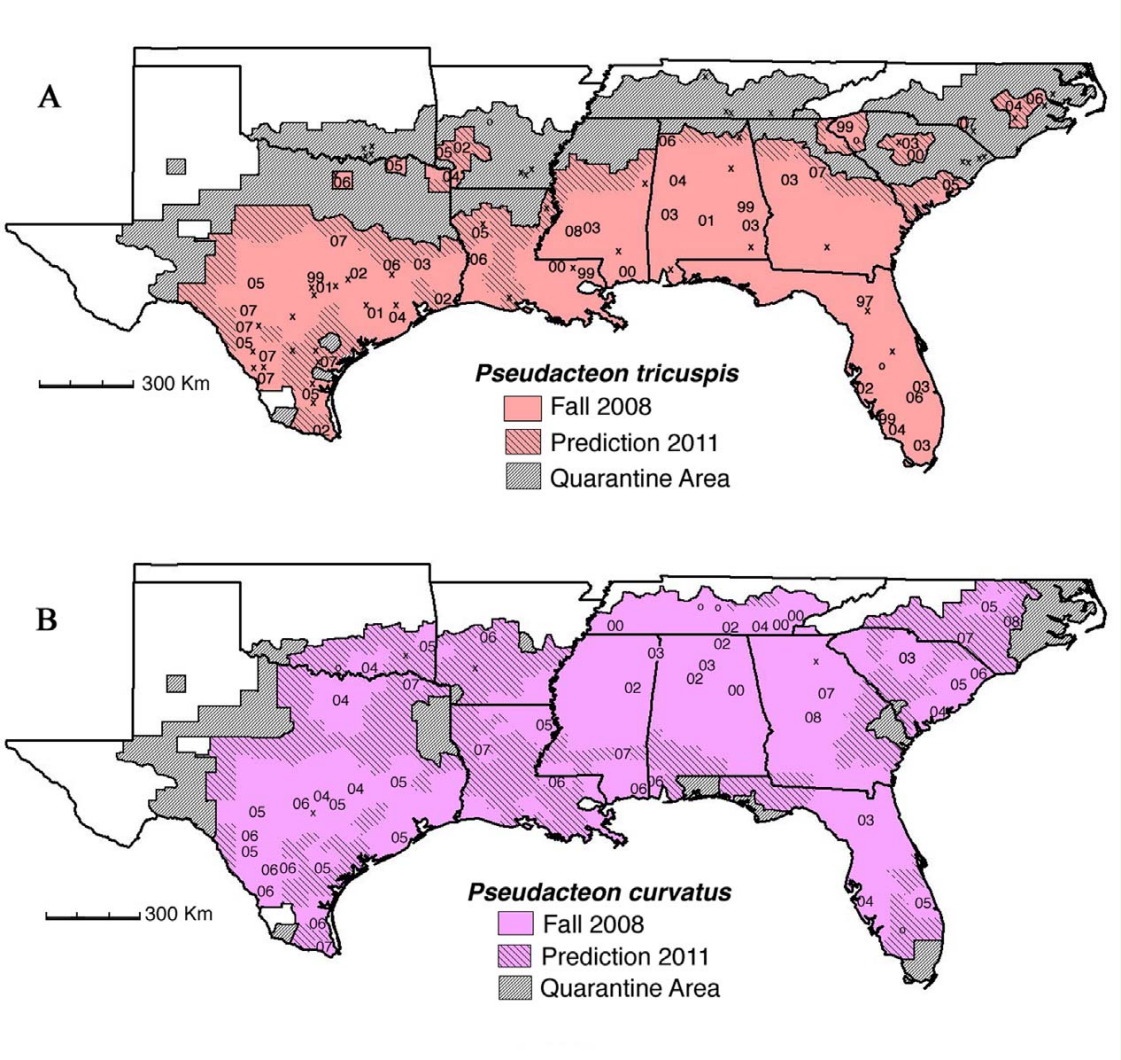
Predicted distribution of the fire ant decapitating flies, *Pseudacteon tricuspis* (A) and *Pseudacteon curvatus* (B) after three years (fall of 2011). Locations and dates of successful releases are indicated by two-digit years. Locations of unsuccessful releases are indicated by a small “x”. Several locations with releases of undetermined status are shown with an “o”. The quarantine area has been expanded to include 29 counties in Texas added to the quarantine area in late 2009. Several additional counties have also been added in Oklahoma, Arkansas, and North Carolina that are likely to be added by 2011. Potential expansion in other states is not predicted, although it is likely to be relatively slow because of cold winter temperatures (Korzukhin et al. 2001). Only the earliest release date is shown for areas with multiple successful releases (e.g. Gainesville, FL and Austin, TX). See Appendices 1–2 for more complete information about field releases. High quality figures are available online.

Estimates for ‘probable’ distributions of flies in the fall of 2008 (Figure 2) and ‘predicted’ distributions in three years time (Figure 3) were based on the confirmed locations in Figure 2 and historical dispersal rates reported in the literature (Porter et al. 2004; Pereira and Porter 2006; Henne et al. 2007a; LeBrun et al. 2008; Porter 2010). Generally, the literature indicates that established populations of decapitating flies expand outward at the rate of about 30 km/yr (Porter 2010); however, this can be quite variable. Newly established populations will usually only expand a few kilometers in the first year and sometimes there is a latency period of several years before any expansion occurs (Porter et al. 2004; Henne et al. 2007a). Expansion rates generally accelerate over time as larger populations make long-distance dispersal events more common (Pereira and Porter 2006; Henne et al. 2007a). In some instances, expansion can be very slow in one direction but rapid in another (Porter et al. 2004; Henne et al. 2007a; LeBrun et al. 2008) with rates in excess of 50 km/yr having been reported (Pereira and Porter 2006). Expansion rates for *P. tricuspis* have been slow in drier portions of Texas (Gilbert et al. 2008) and cooler portions of the range (e.g. the population released in 1999 near Clemson in western South Carolina; Figure 2). High densities of *P. curvatus* may reduce *P. tricuspis* populations in some areas (Porter 2010), most likely slowing rates of *P. tricuspis* expansion (LeBrun et al. 2009). Expansion rates of *P. curvatus* generally appear to exceed those of *P. tricuspis* probably because of shorter latency periods and higher densities (Porter 2010) due to higher densities of the small workers that can serve as hosts for this fly.

In making estimates for the 2008 distributions we tried to take all of the factors above into consideration. Generally, 30 km/yr was used as the estimated rate of expansion for well established populations; however, this was adjusted to match confirmed observations in neighboring counties. Estimates for future distributions were based primarily on observed regional and local rates of expansion (Figure 2). We attempted to be conservative in estimates of current distributions and predictions for future distributions.

Specifically to mitigate over-estimates, counties were generally not considered ‘probable’ in 2008 (Figure 2) or ‘predicted’ in 2011 (Figure 3) until half or more of the county was considered to be occupied by flies.

The uncertainty associated with fly dispersal is one reason why we chose to present distribution results and area estimates at the county level. The other reason is that on a large geographic scale, county-level data is probably sufficiently accurate. We recognize that fire ant populations, and therefore the associated phorid fly populations, may not be equally distributed over a county, especially in arid regions such as west Texas, and along the leading edge of the fire ant infestation. Overall, we feel that a compilation of distribution data on this large geographic scale dictates that we use a fairly large data point in order to graphically illustrate the data, and therefore county-level data points were chosen.

### Observed and predicted fly distributions

*Pseudacteon tricuspis*. Establishment and spread of *P. tricuspis* has been most successful in the southern areas of the imported fire ant range in the United States (Figure 2A) with at least moderate levels of precipitation (>100 cm/year). Field releases at sites in the more northern areas (north of the line of latitude marking the Louisiana border) were only 32% successful (13/40; Appendix 1, Figure 3A) compared to 69% successful (31/45) in the moist southern areas from College Station, Texas and eastward. Releases at sites in the hotter and drier parts of Texas west and south of College Station were intermediate with a 50% success rate (16/32, Appendix 1), a figure that would be lower if multiple releases at the same site were considered separately. Furthermore, it appears that expansion rates of *P. tricuspis* flies released in cool, dry regions were often slower than those released in warm, moist locations (Figure 2A, Figure 3A).

In the fall of 2008, eleven years after the first successful release, *P. tricuspis* occupied about 709,000 km^2^ or about 50% of the fire ant quarantine area (Figure 2A). In three more years, we estimate that this percentage will increase to almost 65% (Figure 3A). In addition to the distributions and releases shown in Figures 2A and 3A, *P. tricuspis* has been released in Puerto Rico and is established on fire ant populations across most if not all of the island. Releases in California were unsuccessful.

***Pseudacteon curvatus*.** Unlike *P. tricuspis, P. curvatus* readily established in northern parts of the fire ant range and expanded rapidly in those areas (Figure 2B; Appendix 2). In the fall of 2008, eight years after the first successful release, *P. curvatus* occupied about 828,000 km^2^ or almost 60% of the fire ant quarantine area (Figure 2B). In three more years, we estimate that this percentage will increase to almost 90% (Figure 3B). The large continuous distribution of *P. curvatus* flies in Tennessee, Mississippi, and Alabama (Figure 2B) is probably almost entirely the result of releases of the Las Flores biotype. While the Las Flores biotype initially established on black and hybrid fire ants, these flies have also expanded southward into areas that are exclusively red fire ants (Shoemaker et al. 1996; Streett et al. 2006; Gardner et al. 2008). Populations of the two biotypes appear to be meeting in the northeast corner of Louisiana and in southern Georgia. We also expect that populations will shortly meet in southern Mississippi and central South Carolina. In addition, *P. curvatus* has been released and established in Puerto Rico but the extent of its distribution there has not been fully determined.

## Discussion

The establishment and natural spread of *P. tricuspis* and *P. curvatus* has been remarkably successful in the southern United States since their original release and establishment in 1997 and 2000, respectively. Comparisons of release methods for *P. tricuspis* (multiple releases over a short period of time vs. multiple releases over longer periods of time) indicate little effect on success rates. However, regional comparisons (north vs. south; moist vs. arid; red fire ants vs. hybrid or black fire ants) suggest *P. tricuspis* is best adapted to warm, moist regions of the southeastern United States. Releases of *P. tricuspis* in the summer were not as successful as in the spring and fall (Figure 1) probably because of high temperatures. The lack of correlation between number of flies released and the success of the *P. tricuspis* releases indicates that factors such as geography, season, and perhaps the habitat of each release site were more important determinants of establishment.

Both biotypes of *P. curvatus* had high establishment rates, with only four confirmed failures using the short term release method, and no failures with the longer term method as well as successes throughout all southeastern areas of the imported fire ant range. Success rates of *P. curvatus* releases were not significantly correlated with season, although releases in the cooler months, November through April, were all successful (Figure 1). The success of *P. curvatus* releases was also not related to the number of flies released, but success on red fire ants was dramatically improved by releasing the Formosa biotype from Argentina (Graham et al. 2003b; Vazquez et al. 2006; Figures 2B, 3B). The high overall success rate with *P. curvatus* is probably because they are naturally better colonizers than *P. tricuspis*, and because they attack minor fire ant workers that are more abundant than majors and therefore occur in higher densities. Although *P. curvatus* was released in higher numbers than *P. tricuspis* and released as larvae in parasitized ants, neither of these factors seemed to improve the success of *P. tricuspis* releases.

By 2011, we expect that *P. tricuspis* will occupy virtually all of the southern ⅔ of the range of imported fire ants in the United States (Figure 3A). Expansion in the northern ⅓ of the range is likely to be gradual based on the poor establishment success and slow expansion rates of *P. tricuspis* flies in northern areas (Figure 3A; Appendix 1). Also, there is reason to expect that the biotype of *P. tricuspis* collected from red fire ants in Jaguariuna, Brazil may not do well on black or hybrid fire ants (Porter and Pesquero 2001; He and Fadamiro 2009). High densities of *P. curvatus* in regions occupied by this species (LeBrun et al. 2009; Porter 2010) may limit the abundance and dispersal rates of *P. tricuspis* probably because attacking flies quickly trigger ant defensive responses which limit the access of other congeners (LeBrun et al. 2009). It would also be interesting to use molecular techniques to assess the fate of several biotypes of *P. tricuspis* released in Texas (Barr and Calixto 2004; Gilbert et al. 2008). Biotypes collected in drier regions of Argentina and from fire ant source populations in Argentina which are more similar to *S. invicta* populations in the United States (Caldera et al. 2008) may be able to out-compete biotypes from wetter regions or biotypes from more distantly related *S. invicta* populations.

*Pseudacteon curvatus* has expanded rapidly throughout the range of imported fire ants in the southeastern United States (Figures 2B, 3B). By 2011, we predict that *P. curvatus* will occupy almost 90% of this area and by 2013 it will occupy essentially all of the quarantined area in the Southeast. Even though *P. curvatus* was first released more than three years after *P. tricuspis, P. curvatus* has expanded its range more rapidly (Porter 2010) perhaps because they occur in much higher densities, at least in Florida and Texas (LeBrun et al. 2009; Porter 2010). *Pseudacteon curvatus* flies have also done very well in northern areas of the range where cold temperatures and black and hybrid fire ants appear to be limiting the success of *P. tricuspis* (Figure 2, 3).

It is interesting that *P. curvatus* flies from black fire ants (Las Flores biotype) have apparently been able to adapt to red fire ant populations in southern Mississippi, Alabama, and Georgia (Figure 2B) even though repeated releases on red fire ants in Florida failed (Graham et al. 2003b). Notably, this biotype was reared for more than three years on red fire ants in the lab before being successfully released on black fire ants in the field (Graham et al. 2003b). Furthermore, the black fire ant (*S. richteri*) and the red fire ant (*S. invicta*) are very close sister species as is evidenced by their ability to form fertile hybrid populations in the United States (Shoemaker et al. 1996). As predicted, both *P. curvatus* biotypes have so far failed to establish on the native fire ant *Solenopsis geminata* (Porter 2000; Vazquez and Porter 2005; unpublished data, S.D.P. and L.E.G.) just as native *Pseudacteon* decapitating flies that attack *S. geminata* have failed to establish on the introduced *S. invicta* populations (Porter and Gilbert 2004).

We expect that, as the large population of *P. curvatus* from black fire ants (Las Flores biotype) in Tennessee, Mississippi, Alabama, and Georgia collides with *P. curvatus* populations from red fire ants (Formosa biotype) released in other states, the Formosa biotype will begin displacing the Las Flores biotype in regions with red fire ants (i.e. southern Mississippi, Alabama, Georgia). We predict this displacement because the two biotypes are probably genetically better adapted to their natural hosts based on their host preferences and the failure of Las Flores biotype to initially establish on red fire ant populations (Porter and Briano 2000; Graham et al. 2003b). Consequently, we also predict that the expansion of a better adapted Formosa biotype into areas where the Las Flores biotype currently attacks red fire ants will result in higher densities of *P. curvatus* than currently exist in those areas. The expansion of Africanized honey bees throughout South and Central America is an example of one biotype of honey bee largely displacing a resident biotype through introgressive hybridization (Winston 1992). In fact, the displacement of black fire ants and the hybrid from southern Mississippi (Streett et al. 2006) is also likely a result of introgressive hybridization. Normal hybrid fly populations between the Las Flores biotype and the Formosa biotype are also a likely result of the colliding fly populations, especially in areas with hybrid fire ants. Future studies of fly population genetics will hopefully test these and other hypotheses.

Strategic selection of future release sites, as well as additional surveys in selected areas, will hopefully fill gaps in the distribution of *P. tricuspis*. Releases of *P. tricuspis* in west Texas and in northeast Texas would supplement adjacent populations that are naturally spreading into those areas. However, additional releases of *P. tricuspis* in the more northern areas where establishment has been limited and expansion rates are very slow may not be the best use of resources considering the need to release additional species of flies already approved for field release (i.e. *P. obtusus and P. cultellatus*). Strategic releases of *P. curvatus* would also help fill gaps in the distribution (Figure 3B), but the best strategy may be to use these temporary gaps to release additional *Pseudacteon* species before *P. curvatus* arrives and interspecific competition makes establishment more difficult (see LeBrun et al 2009; S.D.P., unpublished data; Plowes and L.E.G., unpublished data). Overall, it is clearly time to shift rearing and release efforts to *P. obtusus* (already in mass production in Gainesville, FL and Austin, TX) and *P. cultellatus* (approved for field release, April 2010).

*Pseudacteon tricuspis* and *P. curvatus* are the first classical biological control agents to be successfully established against invasive ants. The release of an ichneumonid parasitoid in New Zealand against vespid wasps (Read et al. 1990) is the only other successful release of a biocontrol agent that we are aware of against a social insect pest. The successful establishment and spread of these two species should encourage exploration and testing of additional natural enemies that can be safely released against social insect pests.

## Figures and Tables

**Appendix 1.**
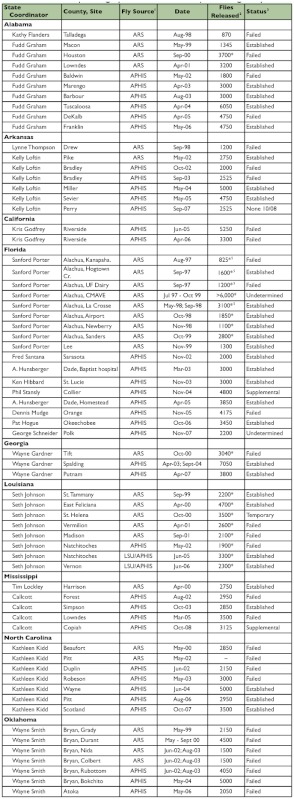
Field releases of the fire ant decapitating fly *Pseudacteon tricuspis* arranged by state and date.

**Appendix 2.**
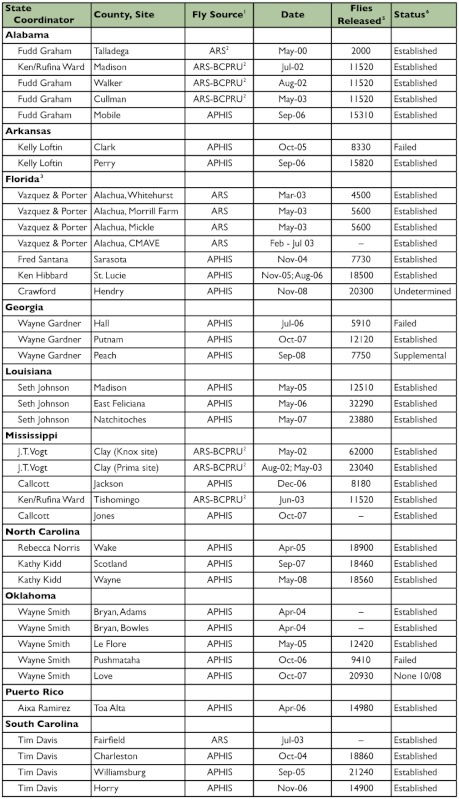
Field releases of the fire ant decapitating fly *Pseudacteon curvatus* arranged by state and date.
